# Macrophage membrane-coated nanoparticles in inflammatory diseases: from bioinspired design to translational potential

**DOI:** 10.1186/s12951-025-03921-x

**Published:** 2025-12-09

**Authors:** Yuyue Hou, Ruolin Wu, Yan Zhou, Chenru Yin, Yongkang Gai, Dawei Jiang, Keshan Wang, Xiaotian Xia

**Affiliations:** 1 Department of Nuclear Medicine, Union Hospital, Tongji Medical College, Huazhong University of Science and Technology, No.1277 Jiefang Avenue, Wuhan, 430022 Hubei China; 2 Hubei Province Key Laboratory of Molecular Imaging, Wuhan, 430022 China; 3 Key Laboratory of Biological Targeted Therapy, the Ministry of Education, Wuhan, 430022 China; 4 Hubei Academy of Scientific and Technical Information, Wuhan, China; 5 Department of Urology, Tongji Medical College, Union Hospital, Huazhong University of Science and Technology, Wuhan, 430022 China

**Keywords:** Macrophage membrane-coated nanoparticles, Biomimetic nanomedicine, Inflammatory diseases, Immune modulation, Targeted drug delivery, Non-tumor pathology, Hybrid cell membranes, Translational nanotechnology

## Abstract

Inflammatory diseases, including autoimmune, infectious, and metabolic disorders, remain major global health challenges due to their complex pathogenesis and limited therapeutic options. In recent years, bioinspired nanotechnology has emerged as a transformative approach in precision medicine. Among various strategies, macrophage membrane-coated nanoparticles (MΦ-NPs) have gained significant attention for their excellent biocompatibility, immune evasion, and lesion-targeting capabilities. This review systematically outlines the construction strategies, biological characteristics, and functional mechanisms of MΦ-NPs. It details the fabrication process, including core material design, macrophage membrane extraction and functionalization, and efficient membrane–core assembly. The current applications of MΦ-NPs in the diagnosis and treatment of non-tumor inflammatory diseases, such as bacterial and viral infections, rheumatoid arthritis, inflammatory bowel disease, atherosclerosis, and bone tissue repair are comprehensively reviewed. Special emphasis is placed on their unique behavior in inflammatory microenvironments, where they enable site-specific drug delivery, prolonged circulation, and immune modulation, offering innovative solutions to long-standing therapeutic challenges. Furthermore, we discuss the emerging hybrid membrane systems that integrate macrophage membranes with those of red blood cells, platelets, or tumor cells, enhancing both targeting and therapeutic performance. Despite promising preclinical results, challenges remain regarding large-scale production, clinical translation, and biosafety evaluation. By integrating insights from nanotechnology, immunology, and translational medicine, this review provides a comprehensive perspective on the design, functionality, and therapeutic frontiers of MΦ-NPs. Continued advances in biomimetic nanomedicine are expected to drive the development of next-generation anti-inflammatory therapies with high precision and minimal systemic toxicity.

**Graphical abstract:**

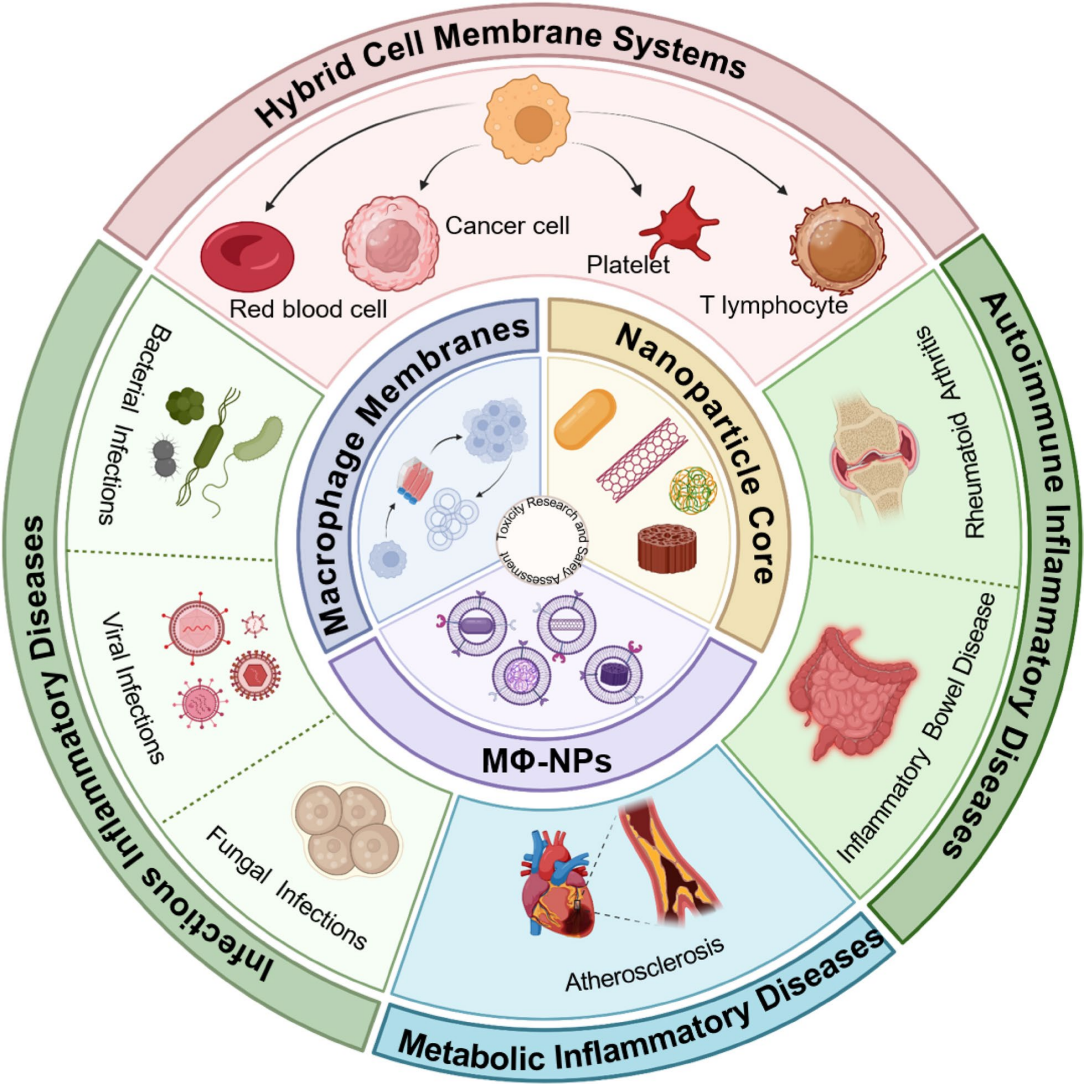

## Introduction

Inflammatory diseases represent a growing global health burden, contributing significantly to morbidity and mortality worldwide [1]. These disorders are characterized by acute or chronic immune activation, driven by internal dysregulation or external insults, ultimately resulting in tissue injury and organ dysfunction. Although inflammation is a fundamental host defense mechanism against infection, injury, or abnormal cellular stimuli, dysregulated or prolonged inflammatory responses can become pathological. This process involves the recruitment and activation of immune cells, such as macrophages and neutrophils, as well as the release of pro-inflammatory cytokines and mediators, including tumor necrosis factor-alpha (TNF-α), interleukin-6 (IL-6), and vascular endothelial growth factor (VEGF) [2].

Persistent inflammation has been implicated in the pathogenesis of numerous diseases, including autoimmune and autoinflammatory conditions, neurodegenerative disorders, cardiovascular system diseases and cancer [3–6]. A wide range of triggers, including microbial infections, hormonal imbalances, environmental toxins, and particulate matter, can initiate and sustain inflammatory cascades [7]. Notably, chronic inflammation can induce genomic instability and promote tumorigenesis. For example, virus-induced inflammation is responsible for approximately 20% of all malignancies, with chronic infections such as hepatitis B and C viruses significantly contributing to liver cancer incidence [2].

Current treatment strategies for inflammatory diseases primarily rely on pharmacological interventions, including glucocorticoids, non-steroidal anti-inflammatory drugs (NSAIDs), and immunosuppressants such as methotrexate and azathioprine [8, 9]. While these agents can effectively suppress inflammation through mechanisms like cyclooxygenase-2 (COX-2) inhibition or immune modulation, their long-term use is frequently associated with adverse effects, such as gastrointestinal toxicity, cardiovascular complications, drug resistance, increased risk of infections, and systemic immunosuppression [2].

In recent years, nanoparticles (NPs) have emerged as versatile tools in biomedical research, offering enhanced drug loading capacity, controlled release, and the ability to integrate multiple therapeutic or diagnostic modalities. NPs have been widely investigated for drug delivery [10], imaging [11], immunomodulation [12], photothermal [13] and photodynamic therapy [14], nucleic acid delivery [15], tissue engineering [16], implantable systems [17] and radiosensitization [18]. Their advantages include enhanced protection of bioactive agents from degradation [19], targeted delivery through surface ligand modification [20], tunable release kinetics via polymer engineering [21], and scalable manufacturing processes [22]. However, clinical translation of NPs remains hindered by several physiological barriers, including rapid clearance by the mononuclear phagocyte system, immunogenicity, and limited penetration within complex inflammatory microenvironments [23]. Achieving sustained and site-specific delivery to inflamed tissues remains a critical challenge.

To overcome these obstacles, biomimetic nanotechnologies, particularly those employing natural cell membrane coatings, have garnered increasing attention. This strategy leverages the functional components of biological membranes to confer NPs with immune evasion, extended systemic circulation, and inflammation-specific targeting. A variety of membrane sources have been explored for this purpose, including red blood cells [24], leukocytes [25], platelets [26], stem cells [27], tumor cells [28], and bacteria [29]. Among these, macrophage membranes stand out due to the innate involvement of macrophages in inflammatory regulation, pathogen defense, tissue remodeling, and tumor immunity [30]. Coating NPs with macrophage-derived membranes not only enhances their biocompatibility and circulatory stability but also improves active homing to inflamed sites via membrane-bound receptors and adhesion molecules.

Macrophages are white blood cells that recognize, engulf, and remove cell debris, pathogens, and abnormal cells. They possess a natural ability to be recruited to inflammatory sites within the body [31, 32]. Macrophage targeting behavior can be summarized in four key steps: chemotaxis, adhesion, phagocytosis, and polarization. During chemotaxis, lesions release chemokines such as CCL2, CCL3, and MCP-1, which bind to CCR2 or CCR4 receptors on macrophages, guiding their migration along concentration gradients. In the adhesion and transendothelial migration stages, initial rolling is mediated by E-selectin and its ligand PSGL-1, followed by firm adhesion via ICAM-1/VCAM-1 binding with integrins α4β1 (VLA-4) and Mac-1 [33]. In the subsequent phagocytosis and polarization stages, macrophages recognize damage-associated molecular patterns (DAMPs) through receptors such as TLR2, TLR4, RAGE, and SR-A/CD36, leading to activation and polarization toward either a pro-inflammatory (M1) or anti-inflammatory (M2) phenotype, which ultimately determines the outcome of inflammation.

**Fig. 1:**
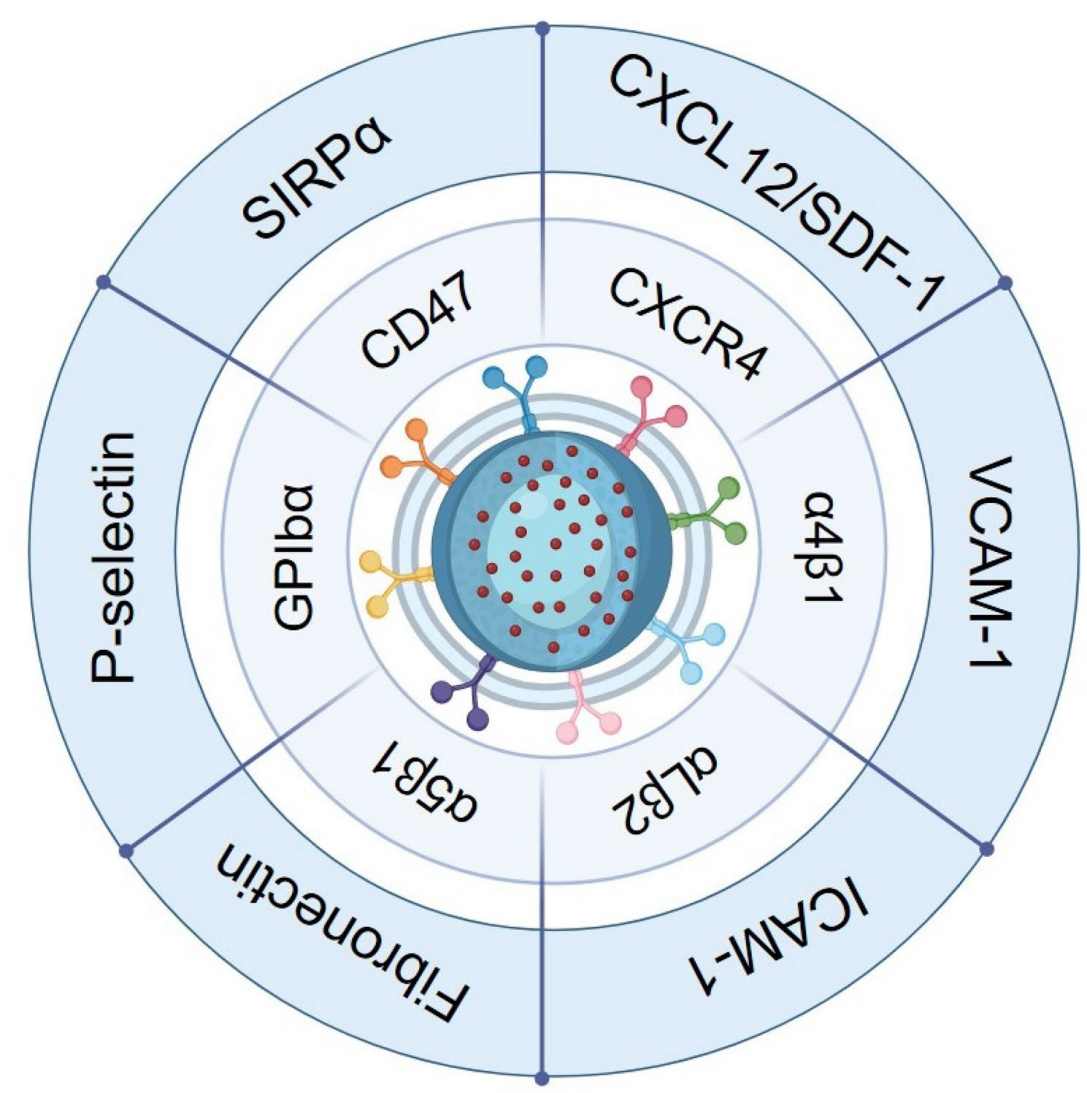
Schematic diagram of key receptors and ligands mediating the inflammatory targeting of MΦ-NPs. The illustration highlights representative receptor–ligand interactions involved in macrophage chemotaxis, adhesion, and immune modulation, including CD47–SIRPα, CCR2–CCL2, and integrin–VCAM/ICAM pathways. The image was created using BioRender.com and is used with permission

Based on these biological properties, macrophage membrane-coated nanoparticles (MΦ-NPs) have emerged as an innovative biomimetic delivery platform with unique advantages for inflammatory disease therapy. By inheriting the surface proteins and biological functions of macrophage membranes, MΦ-NPs retain natural inflammatory tropism, enabling targeted accumulation at lesion sites, neutralization of inflammatory mediators, evasion of reticuloendothelial clearance, and prolonged systemic circulation [34, 35]. Their mechanisms of action encompass four aspects: (i) precise homing to inflamed tissues through membrane adhesion molecules such as ICAM-1, VCAM-1, and PSGL-1 [33, 36–38]; (ii) immune evasion by masking the “exogenous” identity of NPs, thereby reducing phagocytic clearance; (iii) direct adsorption and neutralization of pro-inflammatory cytokines via surface receptors such as TNF-αR and IL-1R [39]; and (iv) controlled release of encapsulated agents—including anti-inflammatory drugs, antioxidants, or microRNAs—in response to microenvironmental stimuli such as pH, temperature, or enzymatic activity [40] (Fig. 1).

Against this backdrop, MΦ-NPs have emerged as a promising class of nanotherapeutics for targeting inflammatory diseases. This review provides a comprehensive overview of their design principles, fabrication techniques, and mechanisms of action, while highlighting their recent applications in the diagnosis and treatment of non-neoplastic inflammatory disorders. Through interdisciplinary analysis, the review establishes an integrated understanding of the design–function–outcome paradigm of MΦ-NPs and discusses forward-looking strategies to address current technological bottlenecks. Continuous innovation in biomimetic nanomedicine is expected to accelerate the clinical implementation of next-generation anti-inflammatory therapies, offering safer and more precise treatment options for patients with inflammatory diseases.

## Macrophage cell membranes-coated nanoparticles

### Construction method

**Fig. 2:**
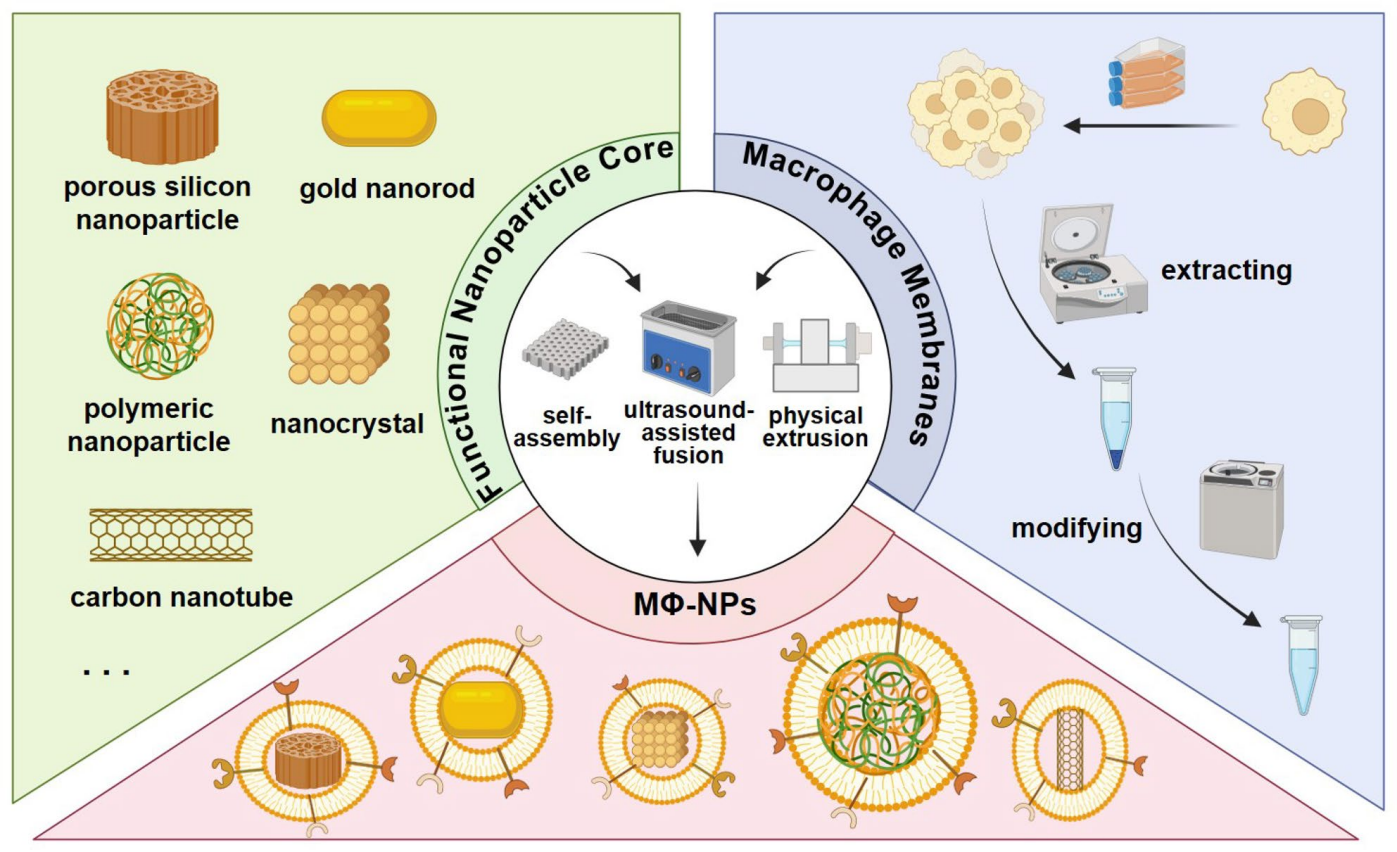
Illustration of the synthesis scheme for macrophage membrane-coated nanoparticles (MΦ-NPs). The figure depicts the general process of MΦ-NPs fabrication, including nanoparticle core design, macrophage membrane isolation and functionalization, and core–shell assembly. The image was created using BioRender.com and is used with permission

The fabrication of MΦ-NPs generally involves three essential stages: (i) the rational design of the functional nanoparticle core, (ii) the isolation and functionalization of macrophage-derived membranes, and (iii) the membrane-to-core assembly process (Fig. 2). The three stages are interrelated and mutually reinforcing, collectively determining the physicochemical stability, biological functionality, and therapeutic efficacy of the final construct (Table 1).

**Table 1:** Overview of the preparation and coating methods for MΦ-NPs

Membrane source	Membrane modification	Nanoparticle core	Combination drugs	Assembly method	Disease model	The role of MΦ-NPs	References
Macrophages	PD-1	PLGA	Rapamycin	Co-extrusion	Glioblastoma	Can cross the BBB, respond to tumor microenvironment recruitment, and accumulate nanoparticles at the tumor site. Due to enhanced infiltration of CD8^+^ CTL, the immune response is strengthened.	[41]
Macrophages	-	Liposomes	DOX and tetrandrine	Self- assembly and co-extrusion	Breast cancer	The system combines DOX and tetrandrine to block DOX efflux, increase DOX enrichment at tumor sites, and reverse DOX resistance. The liposomes exhibit high targeting capability and biocompatibility under the camouflage of macrophage membranes.	[42]
Macrophages	-	Nanowired-Si	-	-	Sepsis	It can rapidly detect extremely low levels of bacteria in blood (1.5 h). It simultaneously identify Gram-type(s) bacteria captured using fluorescent Gram staining.	[43]
Macrophages-derived micro-vesicle	-	Au-CDs	DOX	“Step by step” inter-particle assembled	Lung metastatic breast cancer	Effectively targeting tumor sites and combining with the decomposition reaction of nanocomposites achieves the goals of high therapeutic efficacy and low side effects.	[44]
Macrophages	TLR4	PLGA	Tasquinimod	Co-extrusion	Inflammatory bowel disease	Specifically enhances the accumulation of nanoparticles at sites of intestinal inflammation in mice. Multiple inflammatory factors are adsorbed via the receptors abundant on the surface of MM, thereby modulating and suppressing local inflammation, and reducing the expression of inflammatory signaling pathways and cytokines.	[45]
Cancer cells	-	Upconversion nanoparticles	-	Co-extrusion	Triple-negative breast cancer	The probe possesses homologous targeting and immune evasion capabilities.	[47]
Macrophages	HER2	PLGA	DOX	-	Breast cancer	It targets HER2 + cancer cells and specifically induces suffix-mediated cell therapy, enhancing the efficacy of chemotherapy, working synergistically with cell therapy, and significantly improving anti-tumor effects.	[48]
Macrophages	*P. gingivalis* stimulated	PtNCs	-	Co-extrusion	Alzheimer’s disease	It can significantly inhibit the growth of *P. gingivalis in vitro*, effectively deliver and retain at the infection site in the mouse brain, reduce bacterial load and neuronal damage, thereby improving AD-like cognitive dysfunction in mice with chronic periodontitis.	[49]
Macrophages	-	-	Branched polymer PBAEs (P3)	Self-assembled	Triple-negative breast cancer	It demonstrates high efficacy in killing breast cancer cells and activating anti-cancer immunity. The combination therapy significantly inhibits the growth and metastasis of breast tumors by remodeling the tumor microenvironment and activating immune responses.	[51]
Macrophages	-	Fe_3_O_4_	-	Ultrasound-assisted fusion induced	Atherosclerosis	It can effectively target early atherosclerotic lesions (foam cells) as a contrast agent.	[52]
Macrophages	-	Liposomes	Rg3 and PNS	Co-extrusion	Ischemic stroke	It can avoid single-cell phagocytosis, actively bind to inflammatory endothelial cells, and possesses the ability to cross the blood-brain barrier. It can specifically target ischemic sites, including microglial cells, increase the accumulation of drugs in the brain, improve the cerebral inflammatory environment, and reduce the infarct size.	[53]

*PD-1 Programmed cell death-1; *BBB* Blood − brain barrier; *PLGA* Polylactic acid - glycolic acid copolymer; *CTL* Cytotoxic T-lymphocyte; *DOX* Doxorubicin; *CDs* Carbon dots; *HER2* Anti-human epidermal growth factor receptor-2; *TLR4* Toll-like receptor 4; *PtNCs* Platinum nanoclusters; *Rg3* Ginsenoside Rg3; *PNS* Panax notoginseng saponins

The nanoparticle core serves as the carrier platform, offering controlled drug release, intrinsic biocompatibility, and fundamental targeting capabilities. Frequently utilized materials include biodegradable polymers (e.g., PLGA) [41], biomimetic liposomes [42], and inorganic nanomaterials such as mesoporous silica [43] and gold nanorods [44]. Each class presents distinct advantages: polymers enable sustained drug release and are enzymatically degradable [45]; liposomes closely mimic biological membranes and exhibit low systemic toxicity; inorganic cores exhibit unique physical properties suitable for photothermal or imaging applications. Selecting the optimal nanoparticle core according to specific experimental objectives is critical to achieving desired therapeutic outcomes.

The macrophage membrane is characterized by a phospholipid bilayer enriched with surface proteins essential for cellular recognition, adhesion, and immune modulation. Membrane extraction typically proceeds via two sequential steps: cell membrane lysis followed by purification under mild conditions to preserve native protein conformation and bioactivity [46]. Differential ultracentrifugation, often in combination with density gradient separation, remains the most commonly employed method to isolate intact membrane fragments, despite its limitations in throughput and efficiency [47]. Depending on experimental goals, additional functional modifications may be introduced, such as PD-1 antibody engineering to enhance immunotherapeutic synergy [41], gene editing to overexpress targeting ligands [48], or bacterial pre-stimulation to improve pathogen adhesion and immune activation [49]. In recent years, several commercial kits for macrophage membrane extraction have become available, offering standardized and convenient alternatives for obtaining high-quality membrane materials [50].

The final step, membrane-core assembly, is critical for achieving uniform surface coverage and preserving biological activity. Various techniques have been employed, including self-assembly driven by electrostatic or hydrophobic interactions [51], ultrasound-assisted fusion induced by acoustic cavitation [52], and mechanical extrusion, which facilitates tight wrapping, especially for rigid or anisotropic nanocores [53]. Each method offers specific advantages in terms of coating stability, production scalability, and preservation of membrane orientation.

Although the current development of MΦ-NPs remains largely at the laboratory scale, challenges in reproducibility, scalability, and batch-to-batch consistency persist. Even under small-scale conditions, precise optimization of formulation parameters is required to mitigate heterogeneity. Therefore, standardized quality control protocols are essential to ensure reproducibility, functional integrity, and translational viability.

### Characterization

Comprehensive characterization of MΦ-NPs encompasses both physicochemical profiling and biological validation, which collectively confirm successful membrane integration and the preservation of native biofunctions.

Dynamic light scattering (DLS) typically reveals a modest increase in hydrodynamic diameter and a shift in zeta potential post-coating. The particle size increase typically ranges from 5 to 20 nm [13, 54], though membrane-induced steric repulsion can also reduce aggregation, occasionally resulting in a size decrease of approximately 30 nm [55]. The zeta potential generally approaches that of native macrophage membrane vesicles, indicating successful surface modification [32, 35]. Transmission electron microscopy (TEM) provides visual confirmation of the membrane coating, often revealing a distinct corona surrounding the nanoparticle core.

**Fig. 3:**
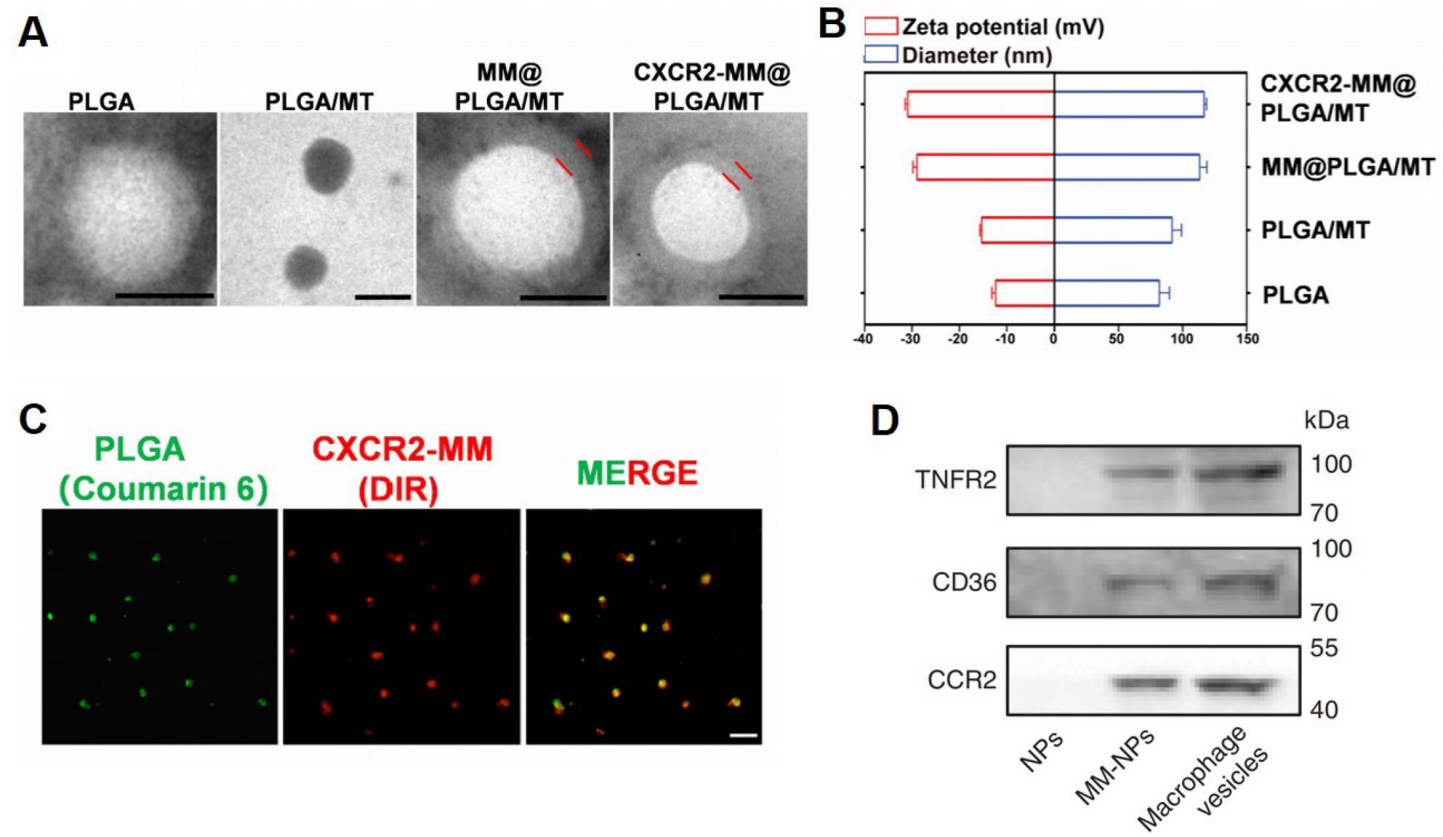
Characterization of MΦ-NPs. (**A**) Representative TEM images of PLGA, PLGA/MT, MM@PLGA/MT, and CXCR2-MM@PLGA/MT nanoparticles. Scale bar = 100 nm. (**B**) Average particle diameter and ζ-potential analysis of CXCR2-MM@PLGA/MT, MM@PLGA/MT, PLGA/MT, and PLGA nanoparticles. (**C**) Fluorescence colocalization of the RAW 264.7-CXCR2-MM membrane shell (red) and PLGA core (green). Scale bar = 20 nm. (**D**) Western blotting of protein markers comparing nanoparticle formulations, macrophage membrane-derived vesicles, and MM-NPs. Figure adapted from Long Yang et al. [39] and Cheng Gao et al. [57], with permission

For biological validation, protein-based assays are frequently employed. Western blotting is used to detect the presence of key membrane proteins retained on the nanoparticle surface [56], while SDS-PAGE facilitates comparative analysis between source cell membranes, extracted vesicles, and the final MΦ-NPs product. These analyses collectively ensure the structural and functional fidelity of the membrane coating, as well as the effective biomimicry essential for in vivo performance (Fig. 3). However, it remains technically challenging to accurately determine the proportion of functional regions preserved on the extracted membrane surface. While SDS-PAGE and related assays confirm protein presence, they cannot assess membrane protein orientation, which critically influences biological functionality. Addressing this gap will require multidimensional analytical techniques, such as cryo-electron tomography or high-resolution surface plasmon resonance mapping, to systematically elucidate membrane topology and guide the rational design of future MΦ-NPs.

Currently, physicochemical and biological characterization constitute the core of MΦ-NPs quality control. While these methods can effectively assess fabrication success and biofunctionality, the absence of standardized evaluation criteria limits cross-study reproducibility. Developing unified operational protocols and quantitative benchmarks for MΦ-NPs quality assurance will be essential for advancing their clinical translation.

### The typing characteristics and applications of macrophage cell membranes

Macrophage membranes can be functionally classified according to their polarization state, most notably the pro-inflammatory M1 phenotype and the anti-inflammatory M2 phenotype, each exhibiting distinct biological and therapeutic roles.

Membranes derived from M1-polarized macrophages exhibit robust pro-inflammatory activity and active homing capabilities [58], rendering them advantageous for targeted delivery of therapeutic or imaging agents to inflamed or tumor-associated sites [59]. Moreover, M1-derived vesicles have been demonstrated to reprogram tumor-associated macrophages (TAMs) toward a more immunostimulatory M1-like phenotype [60], thereby enhancing local cytokine production and reactivating suppressed antitumor immunity [61].

In contrast, M2-type macrophage membranes are characterized by their anti-inflammatory properties and reduced immunogenicity. They have shown therapeutic utility in immune modulation and tissue repair, especially in scenarios involving chronic inflammation or immune tolerance, due to their ability to suppress pro-inflammatory cascades in both macrophages and resident stromal cells such as synoviocytes [62].

Together, M1 and M2 membrane-coated nanoplatforms offer phenotype-dependent therapeutic strategies, enabling tailored interventions for diverse pathological microenvironments. Future research should focus on systematically elucidating the phenotypic heterogeneity, signaling pathways, and microenvironmental compatibility of M1/M2 membranes. Selecting the optimal phenotype based on disease context will be crucial for advancing MΦ-NPs from preclinical development to clinical translation.

## Application in non-tumor diseases

With ongoing advancements in nanobiotechnology, MΦ-NPs have emerged as promising platforms for modulating inflammation across a broad spectrum of diseases. While originally developed with oncological applications in mind, recent evidence highlights their efficacy in non-tumor inflammatory contexts. In these settings, macrophage-derived features such as immunotargeting, biocompatibility, and immune evasion provide distinct therapeutic advantages.

This section systematically reviews the preclinical applications of MΦ-NPs in non-malignant inflammatory diseases, emphasizing their functional utility in infectious, autoimmune, and metabolic disorders. By harnessing the immunomodulatory capabilities of engineered macrophage membranes, these nanoplatforms represent a significant advancement in precision nanomedicine for non-cancer inflammatory conditions.

### Infectious inflammatory diseases

Infectious inflammatory diseases represent a substantial subset of immune-related disorders, characterized by immune activation following invasion by exogenous pathogens. These conditions are classically categorized into bacterial, viral, and fungal infections. This section focuses on the emerging diagnostic and therapeutic applications of MΦ-NPs across these categories (Table 2).

**Table 2:** Overview of application in infectious inflammatory diseases

Type of disease	Disease model	Applied nanoparticles	Hydrodynamic size (nm)	Zeta potential (mV)	Application scenarios	Key findings	References
Bacterial infections	Sepsis	Nanowire silicon	-	-	Bacterial detection and Gram classification	Utilizing bacteria-activated macrophage membrane-coated nanowire silicon surfaces enables rapid detection and Gram identification, featuring short detection time while allowing simultaneous diagnosis and treatment.	[43]
	Staphylococcus aureus infection	Triclosan and ciprofloxacin self-assemble into antibacterial nanoparticles	Approximately 100	Approximately − 15	Selectively entering infected macrophages and effectively killing intracellular bacteria	It provides a new strategy for treating intracellular bacterial infections, which is expected to address the challenges of existing antibiotic therapies and offers new directions and approaches for the clinical treatment of persistent infections.	[68]
	Anthrax	PLGA	Approximately 170	Approximately − 15	Stimulate immunity	Utilizing the natural interaction between PA and macrophages to prepare nanotoxoid vaccines, achieving co-delivery of antigens and adjuvants to stimulate long-lasting immunity.	[69]
	*Pseudomonas aeruginosa* infection	Polymeric nanoparticle cores	Approximately 100	Approximately − 30	Polyantigenicity, neutralizing bacterial hemolysis and cytotoxicity, eliciting potent humoral immune responses	MΦ-toxoid can display a broad range of *Pseudomonas aeruginosa* antigens, and this vaccine is capable of eliciting effective immunity against pathogenic *Pseudomonas aeruginosa*. When administered to mice via different routes of vaccination, the nanotoxoid can induce a robust humoral immune response.	[70]
Viral infections	Influenza	PDA	Approximately 220	Approximately − 25	Enhancing delivery efficiency, suppressing cytokine storms, and inhibiting viral replication	Bionic nanoparticles can actively accumulate in lung injury models of virus infection. At the infection site, PDA nanoparticles can scavenge excess reactive oxygen species while being oxidized and degraded to achieve controlled release of oseltamivir phosphate.	[72]
	COVID-19	Polymeric nanoparticle	Approximately 100	Approximately − 25	By identifying host cells infected with SARS-CoV-2.	Both Epithelial-NS and MΦ-NS can neutralize SARS-CoV-2 infectivity in a dose-dependent manner, and the nanosponge platform is insensitive to viral mutations or even viral species. MΦ-NS exhibits superior advantages compared to Epithelial-NS.	[73]
	COVID-19	PLGA	98.6 ± 4.0	−23.1 ± 2.2	Absorb pro-inflammatory cytokines, neutralize SARS-CoV-2 infectivity, and alleviate the “cytokine storm” induced by SARS-CoV-2 infection.	NPs can selectively bind to viruses, inhibit their invasion of host cells, achieve photothermal destruction of viruses under NIR irradiation, and also absorb pro-inflammatory cytokines. TN@AM NPs possess both antiviral and anti-inflammatory functions. The treatment regimen involving nebulized inhalation of NPs combined with NIR irradiation of the respiratory tract can reduce viral transmission.	[74]
	Monkeypox	PLGA	121.9	−32.6	Lesion tracking and fluorescence imaging, photothermal virus elimination, promoting wound healing, and blocking viral transmission	After laser irradiation, the virus is eliminated by the photothermal effect, and the infected wound rapidly heals, successfully blocking viral transmission.	[75]
Fungal infections	*C. albicans* infection	UPNC	35–53	Approximately − 20	Phoxinus targeting and detection	It possesses excellent fungal binding capability, minimal monocyte internalization, high stability, and biocompatibility. Moreover, it emits fluorescence only in the presence of fungal cells, precisely reporting *C. albicans* under near-infrared illumination, and subsequently activating PDT to kill fungal cells. It is suitable for both *in vitro* and *in vivo* fungal diagnosis and treatment.	[76]

*COVID-19 coronavirus disease 2019; *PLGA* polylactic acid-glycolic acid copolymer; UCNPs upconversion nanoparticles; *C. albicans* *candida albicans*; *PDA* polydopamine; *PDT* photodynamic therapy

#### **B**acterial infection

Bacterial infections remain a major global health burden and are the second leading cause of mortality worldwide [63, 64]. Common pathogens such as *Escherichia coli* and *Staphylococcus aureus* typically infiltrate the host via respiratory, gastrointestinal, or dermal routes. These organisms proliferate and secrete virulence factors that trigger innate immune activation and systemic inflammation. Although antibiotics remain the gold standard for treatment, rising antibiotic resistance has created an urgent clinical need for novel therapeutic platforms [65]. Early and accurate diagnosis is critical for improving outcomes [66, 67].

**Fig. 4:**
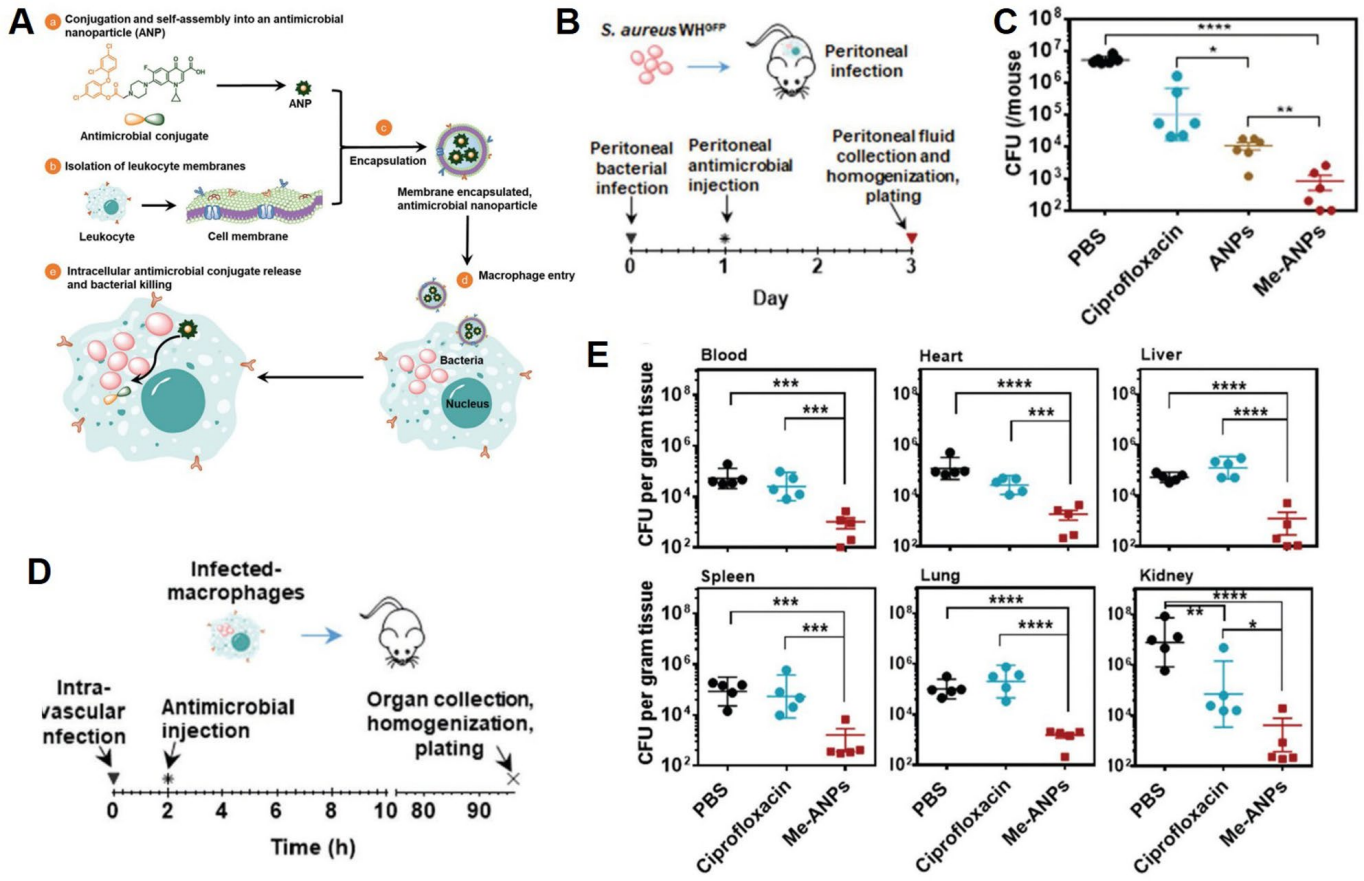
Design and antibacterial efficacy of macrophage–monocyte membrane-encapsulated antimicrobial nanoparticles (Me-ANPs). (**A**) Schematic illustration of Me-ANP design, synthesis, and antibacterial mechanism. (**B**) Mouse acute peritonitis model. (**C**) Bacterial CFU counts retrieved from 5 mL peritoneal fluid 2 days after antimicrobial injection (*n* = 5 per group). (**D**) Mouse intravenous infection model. (**E**) CFU counts per gram of homogenized organ tissue 4 days after intravenous administration (*n* = 5 per group). Data are presented as mean ± SD. Asterisks indicate statistical significance at *p* < 0.05 (*), *p* < 0.01 (**), *p* < 0.001 (***), and *p* < 0.0001 (****), determined by one-way ANOVA. Figure adapted from Yuanfeng Li et al. [68], with permission

Liu et al. [43] designed a macrophage-membrane functionalized microfluidic system using nanowire silicon substrates to enable Gram-type classification and early detection of bacteria within 1.5 h, particularly useful in the context of sepsis. Li et al. [68] developed membrane-encapsulated antibacterial nanoparticles (Me-ANPs) by conjugating triclosan and ciprofloxacin, which preferentially accumulate in infected macrophages and surpass conventional treatments in both in vitro and in vivo infection models (Fig. 4).

Holay et al. [69] fabricated a macrophage membrane-coated nano-toxoid (NT[PA]) vaccine that mimics *Bacillus anthracis* interactions, producing a single-dose immunization capable of eliciting robust humoral and cellular immunity. Similarly, Wei et al. [70] reported a multivalent MΦ-toxoid vaccine targeting *Pseudomonas aeruginosa*, inducing both mucosal and systemic immunity through intranasal or subcutaneous routes.

Collectively, MΦ-NPs represent a transformative platform for combating bacterial infections by enabling early diagnostics, circumventing resistance, and facilitating next-generation vaccine strategies.

#### Viral infection

Viral infections continue to pose significant public health threats, ranging from seasonal influenza to pandemic-scale viruses such as SARS-CoV-2 [71]. Upon host entry, viruses commandeer cellular machinery, often initiating exaggerated inflammatory responses or “cytokine storms”. MΦ-NPs offer multifaceted capabilities including viral neutralization, immunomodulation, and inflammation attenuation.

**Fig. 5:**
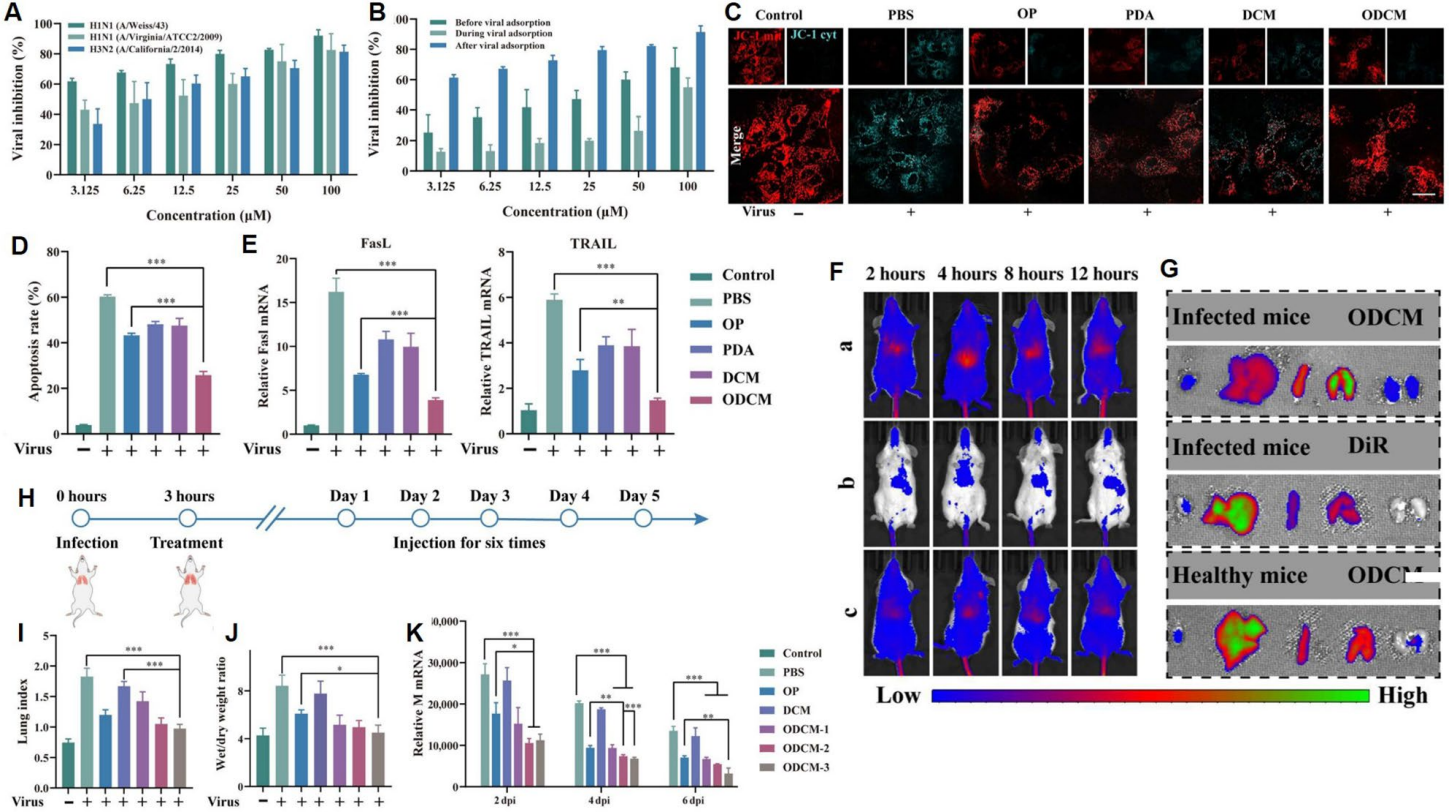
Antiviral effects and biological performance of ODCM nanoparticles. (**A**) Inhibition rate of ODCM at different concentrations against various viruses. (**B**) Viral suppression in infected cells treated with ODCM for 72 h (*n* = 3). (**C**) Mitochondrial membrane potentials (MMPs) in MDCK cells under different treatments. Scale bar = 20 μm. (**D**) Apoptosis rates of virus-infected cells after 48 h of ODCM treatment (**E**) mRNA expression levels of apoptosis-related ligands FasL and TRAIL (*n* = 3). (**F**) In vivo fluorescence imaging at various time points: (a) infected mice after DiR-labeled ODCM, (b) infected mice after DiR only, and (c) healthy mice after DiR-labeled ODCM. (**G**) Ex vivo imaging of major organs (from left to right: heart, liver, spleen, lung, and kidney) after 12 h. (**H**) Therapeutic regimen for virus-infected mice (*n* = 9). (**I–K**) Lung index, wet/dry weight ratio, and M gene mRNA expression across treatment groups. Data are expressed as mean ± SD (*n* = 3–9). *p values* were determined by one-way ANOVA. **p < 0.001. Figure adapted from Na Yin et al. [72], with permission

Yin et al. [72] engineered a nanoparticle system (ODCM) composed of oseltamivir phosphate-loaded polydopamine nanoparticles encapsulated in macrophage membranes. This formulation not only inhibited viral replication but also reduced oxidative stress and improved lung pathology in infected models (Fig. 5). Zhang et al. [73] developed nano-sponges derived from lung epithelial and macrophage membranes capable of neutralizing SARS-CoV-2 across strains, with MΦ-based vesicles showing superior anti-inflammatory potential.

Li et al. [74] introduced TN@AM NPs for photothermal ablation of viruses combined with cytokine adsorption. TBD@M NPs [75], composed of AIE-active molecules encapsulated in MΦ membranes, enabled NIR-II imaging and virus eradication in monkeypox models. These examples underscore the therapeutic flexibility of MΦ-NPs in managing complex viral infections.

#### Fungal infection

Fungal pathogens such as *Candida* and *Aspergillus* predominantly affect immunocompromised individuals. While research remains limited in this domain, MΦ-NPs have demonstrated emerging potential.

Wang et al. [76] constructed multifunctional macrophage membrane-coated nanocomposites incorporating lanthanide-doped upconversion cores, methylene blue, and DNA sensors. These nanoparticles selectively targeted *Candida albicans*, enhanced DNA recognition, enabled photodynamic fungal destruction, and simultaneously attenuated local inflammation with minimal off-target effects.

### Autoimmune inflammatory diseases

Autoimmune inflammatory diseases arise from aberrant immune recognition, wherein the body’s immune system mistakenly targets self-antigens, triggering sustained inflammatory cascades and progressive tissue damage [77]. Conditions such as rheumatoid arthritis (RA) and inflammatory bowel disease (IBD) are prototypical examples that significantly impair quality of life. Conventional therapies often manage symptoms without halting disease progression. The emergence of MΦ-NPs offers new opportunities for disease-modifying interventions with improved precision and reduced systemic toxicity.

#### Rheumatoid arthritis

RA is a chronic autoimmune disorder that primarily affects the synovial joints and is driven by overproduction of pro-inflammatory mediators, notably TNF-α [78–80]. Despite the efficacy of DMARDs, a proportion of patients exhibit inadequate responses, necessitating alternative therapeutic modalities [81].

Shan et al. [82] designed a biomimetic nanocomplex (RCPsT NCs) comprising siTNF-α condensed with a cationic helical polypeptide (PG), co-loaded with catalase (CAT) and coated in macrophage membranes. The construct facilitated prolonged systemic circulation and targeted delivery to inflamed joints. Within the oxidative microenvironment, CAT catalyzed the conversion of H₂O₂ into O₂, triggering membrane disassembly, enhancing siRNA exposure, and promoting TNF-α knockdown, thereby ameliorating inflammation and oxidative stress.

Li et al. [83] introduced a simplified coating strategy using macrophage-derived microvesicles (MMVs) to encapsulate tacrolimus (T-MNPs). These MMVs preserved surface functional proteins while ensuring high colloidal stability and biosafety. The T-MNPs achieved targeted delivery to inflamed synovium, significantly attenuating synovitis, joint swelling, and systemic inflammatory responses.

Zhang et al. [84] proposed a multi-functional macrophage-coated platform integrating photoacoustic probes, NO scavengers, and glucocorticoid prodrugs for RA theranostics. This system offered NO-responsive imaging and therapeutic release, effectively reducing synovial inflammation and promoting repolarization of M2-dominant macrophages toward a pro-resolving M1 phenotype.

#### Inflammatory bowel disease

IBD, encompassing Crohn’s disease and ulcerative colitis, is characterized by chronic mucosal inflammation and immune dysregulation in the gastrointestinal tract [85, 86]. Current treatments remain suboptimal in targeting mucosal sites without systemic side effects.

Duan et al. [87] developed pH-sensitive oral MΦ-NPs (cp-MΦ-NPs) capable of navigating the harsh gastric environment and releasing therapeutics specifically in the inflamed colon. These nanoparticles demonstrated high cytokine-binding capacity and immunomodulatory effects. In murine models, both prophylactic and therapeutic administration markedly reduced disease severity, confirming their potential as site-specific anti-inflammatory agents.

### Metabolic inflammatory diseases

Metabolic inflammatory diseases are intimately linked to systemic metabolic dysfunction and low-grade chronic inflammation. Atherosclerosis serves as a prototypical example where MΦ-NPs provide novel diagnostic and therapeutic solutions.

####  Atherosclerosis

Atherosclerosis (AS), a chronic inflammatory condition, remains the leading contributor to cardiovascular mortality [88–90]. Inflammatory cascades play critical roles in plaque initiation, progression, and rupture. MΦ-NPs offer dual utility in non-invasive imaging and site-specific drug delivery.

A study by Yi et al. [52] introduced Fe₃O₄@M nanoparticles for molecular MRI of early-stage atherosclerotic plaques. These particles exhibited high specificity toward VCAM-1 expressed by dysfunctional endothelial cells, enabling sensitive detection of foam cells with minimal off-target effects.

**Fig. 6:**
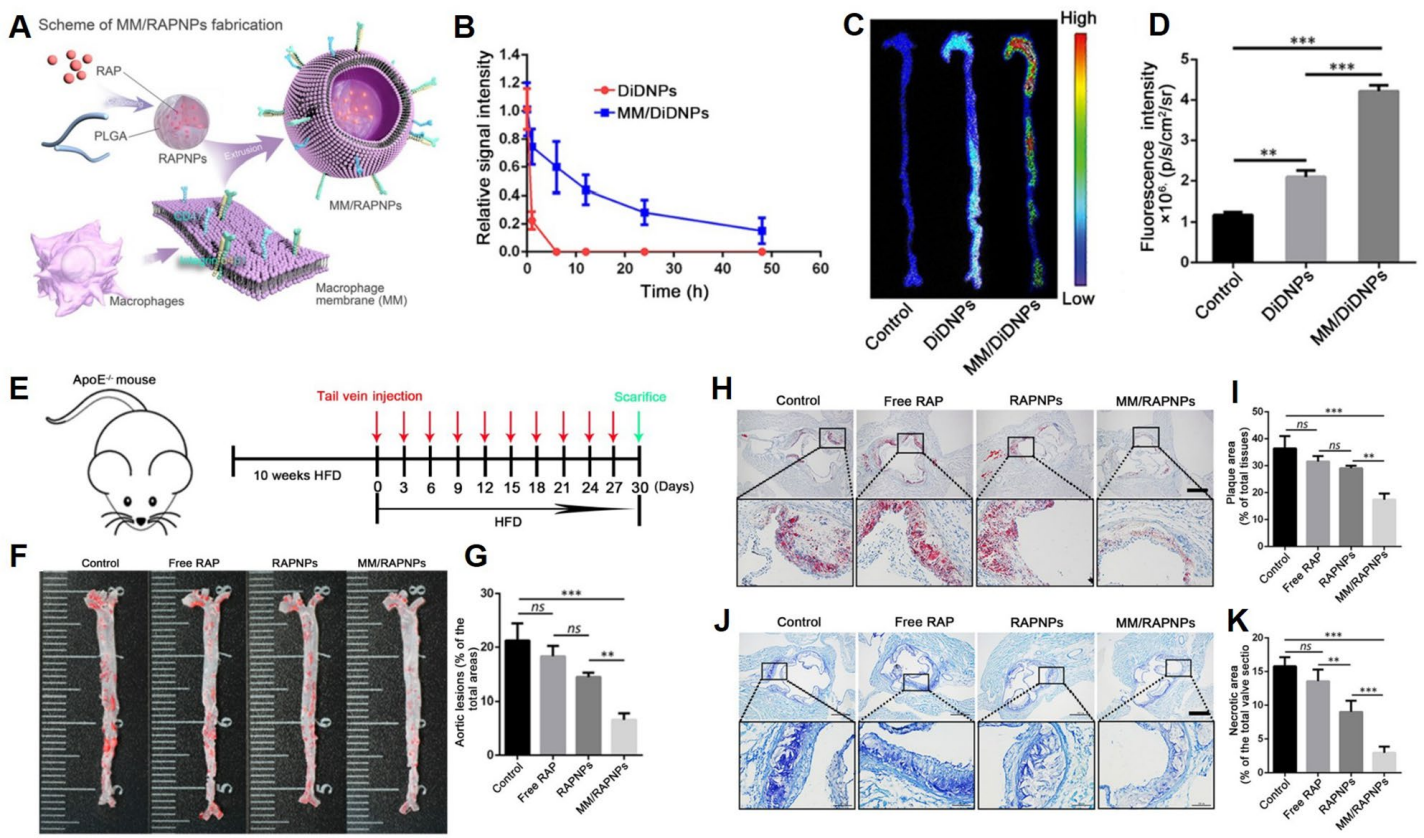
Therapeutic evaluation of macrophage membrane–coated rapamycin nanoparticles (MM/RAPNPs) for atherosclerosis. (**A**) Schematic illustration of MM/RAPNP fabrication and treatment strategy for atherosclerosis (AS). (**B**) Relative fluorescence intensity of DiDNPs and MM/DiDNPs in blood. (**C**) Representative ex vivo fluorescence images and (**D**) quantitative analysis of DiD accumulation in aortic tissues 24 h post-injection (*n* = 3). (**E**) Experimental design overview. (**F**) En face ORO-stained aortas and (**G**) quantitative lesion area analysis (*n* = 5). (**H**) ORO-stained cross-sections of aortic roots. Scale bar = 500 μm. (**I**) Quantitative analysis of lipid deposition area (*n* = 5). (**J**) Toluidine blue–stained necrotic core regions in aortic plaques. Scale bar = 500 μm. (**K**) Quantitative analysis of necrotic core area (*n* = 5). Data are shown as mean ± SD. *p < 0.01, **p < 0.001, ns = not significant. Figure adapted from Yi Wang et al. [50], with permission

Wang et al. [50] constructed MM/RAPNPs, which are rapamycin-loaded PLGA nanoparticles camouflaged with macrophage membranes, for long-term anti-AS therapy. The coating reduced macrophage clearance and directed nanoparticles toward activated endothelial regions, resulting in delayed plaque development and robust safety over prolonged administration (Fig. 6).

Liu et al. [54] formulated MPR NPs by encapsulating Prussian blue nanoparticles and rosuvastatin in macrophage membranes. These particles alleviated HHcy-induced atherosclerosis by inhibiting macrophage pyroptosis and modulating the gut microbiome. Therapeutic efficacy was validated in ApoE⁻/⁻ mouse models, highlighting the potential for metabolic reprogramming and inflammation suppression.

### Others applications

Beyond inflammatory and metabolic diseases, MΦ-NPs have also demonstrated beneficial effects in treating bone defects and fractures. Fractures disrupt the continuity of bone tissue, leading to pain, reduced mobility, and diminished quality of life. Bone-resident macrophage subsets, such as osteoclasts and osteomacs, are actively involved in bone remodeling and healing at every stage [91, 92]. Inspired by these biological roles, MΦ-NPs are now being investigated for their role in regulating the bone regeneration microenvironment.

Yin et al. [16] designed anti-inflammatory nanocapsules encapsulated in macrophage membranes and loaded with resolvin D1 (RvD1). The formulation exhibited synchronized drug release with endogenous bone regeneration timelines and promoted M2 macrophage polarization, accelerating femoral defect repair in murine models.

Wu et al. [93] fabricated sSDF-1α and siCkip1 dual-loaded nanoparticles with reversible macrophage membrane coating. Upon exposure to elevated H₂O₂ levels at fracture sites, the membrane disassembled, releasing the cargo to direct mesenchymal stem cell recruitment and osteogenic differentiation. This approach demonstrated superior healing outcomes, suggesting new directions for bone regenerative therapies.

## Hybrid cell membrane systems

To harness the full biological potential of cell-derived membranes, researchers have increasingly explored hybrid membrane strategies, aiming to integrate the complementary advantages of different cell types. Among these, red blood cells (RBCs), the most abundant and long-lived circulating cells, offer exceptional attributes such as high biocompatibility, intrinsic degradability, and extended systemic circulation. Cao et al. [94] engineered a hybrid membrane by fusing RBCs with macrophage membranes (EMHM) to construct EG@EMHM nanoparticles encapsulating emodin and glycyrrhizic acid. This system enhanced anti-tumor efficacy via photodynamic therapy, improved emodin solubility and bioavailability through glycyrrhizic acid, and significantly prolonged blood circulation through the RBC component.

Ji et al. [95] designed near-infrared-responsive hollow copper sulfide nanoparticles (CuS SF@CMV NPs) cloaked with tumor cell–macrophage hybrid membranes. These particles encapsulated sorafenib and were further functionalized with anti-VEGFR antibodies, endowing them with combined properties of homotypic targeting, immune evasion, and inhibition of tumor signaling pathways. This multifunctional approach synergistically enhanced the efficacy of photothermal and chemotherapeutic treatment in liver cancer models.

Xiong et al. [96] developed siIRF1@ZIF@HM nanoparticles, featuring T lymphocyte–macrophage hybrid membranes and a ZIF-8 core loaded with siRNA targeting IRF1. This design enhanced the targeted delivery to inflamed myocardial tissues and M1 macrophages, while the pH-responsive core ensured efficient intracellular release of siRNA in the pathological environment.

In a parallel effort, Zhou et al. [15] created BSPC@HM nanocomplexes reversibly camouflaged with platelet–macrophage hybrid membranes to deliver Sav1 siRNA for myocardial regeneration. These constructs exploited the inflammatory homing of macrophages and thrombus-targeting of platelets, facilitating selective accumulation in ischemia-reperfusion injured myocardium. Acid-triggered membrane shedding enabled cellular internalization and Hippo pathway inhibition, ultimately promoting cardiomyocyte regeneration. These examples highlight the versatility and synergistic potential of hybrid membranes, which combine the biological functions of distinct membrane types. Despite promising progress in oncology and cardiology, their application in inflammatory disease contexts remains underdeveloped and warrants future exploration.

Based on the complementary properties of various cell membranes, it is reasonable to speculate that hybrid systems combining macrophage and platelet membranes, or macrophage and stem cell membranes, hold substantial potential in autoimmune and vascular inflammatory diseases. The macrophage membrane contributes inflammation-targeting and phagocytic regulation; the platelet membrane provides hemostatic and tissue repair responses; and the stem cell membrane offers immunoregulatory and regenerative signaling. The integration of these functionalities may achieve precise localization to inflamed sites, dynamic immune microenvironment modulation, and enhanced tissue repair. Such hybrid designs represent a promising future direction for the combined treatment of complex inflammatory disorders.

## Recent patents based on MΦ-NPs

With the rapid advancement of MΦ-NPs technology and the continuous expansion of their biomedical applications, the number of related patent filings has grown substantially since 2020. This trend reflects a clear acceleration in the transition from fundamental research toward industrial application.

Table 3 summarizes representative biomimetic patents related to macrophage membrane-coating technologies from 2020 to 2025, compiled from international databases such as the World Intellectual Property Organization and the China National Intellectual Property Administration. These patents encompass innovations across multiple dimensions, including core technological enhancements, optimization of fabrication processes, and expansion into clinical application domains.

**Table 3:** A list of selected patents and patent applications related to MΦ-NPs (Data obtained on 10/26/2025)

Patent/application number	Patent title	Nanoparticle core	Indication(s)	Assignee	Filing year	Status
CN111686251	Bionic nano material for sonodynamic/gas synergistic anti-tumor treatment and preparation method of bionic nano material	AuNPs and silicon	Cancer	Mengchao Hepatobiliary Hospital of Fujian Medical University, Fuzhou, China	2020	Granted
CN111803653	Gene delivery system capable of removing mixed cell membrane coating as well as preparation method and application of gene delivery system	Spiral polypeptide material and polymer material	Targeted delivery	Soochow University, Suzhou, China	2020	Granted
CN111647952	Preparation method and application of cell membrane coated nano topological structure array	Nano topological structure array	Capture of bacteria	Soochow University, Suzhou, China	2020	Granted
CN112438960	An alveolar macrophage-like multifunctional nanoparticle loaded with aggregated luminescent photothermal material and its preparation method and application	PLGA	Virus infection	The Fifth Affiliated Hospital, Sun Yat-sen University, Zhuhai, China	2020	Filed
CN114057818	Nanomedicine for inhibiting vascular intimal hyperplasia and its applications	PCM	Atherosclerosis	Chongqing University, Chongqing, China	2021	Granted
CN113750232	A macrophage membrane-coated arginine deiminase/catalase/IR780 nanoparticle, its preparation method and applications	Arginine deiminase/catalase/IR780 nanoparticles	Cancer	Chongqing Medical University, Chongqing, China	2021	Granted
CN113577316	A biomimetic nanohydrogel coated with macrophage membrane and loaded with manganese dioxide (MnO_2_) and cisplatin (Pt), along with its preparation and application	MnO_2_ and Pt	Glioma	Donghua University, Shanghai, China	2021	Filed
CN115429772	A macrophage membrane-coated manganese dioxide nanoparticle for regulating the cerebral ischemic microenvironment	MnO_2_	Brain inflammation	Fudan University, Shanghai, China	2021	Granted
CN114544930	Method for coating magnetic bead fishing traditional Chinese medicine active ingredients with cell membranes based on electroporation and application of method	BSA-RDG magnetic nanoparticles	Fishing the anti-inflammatory active ingredients in the radix aconiti lateralis praeparata	Chinese People Liberation, Air Force and Military University, Xi’an, China	2022	Filed
CN115487320	Photoacoustic imaging bionic nano-probe material for identifying cervical cancer and preparation method of photoacoustic imaging bionic nano-probe material	Mesoporous silicon	Cervical cancer	The Third Affiliated Hospital of Guangzhou Medical University, Guangzhou, China	2022	Granted
CN115804762	A macrophage membrane-coated ectopic endometrium-targeting nanoparticle, its preparation method, and applications	Drug nanoparticles or drug lipid nanoparticles	Endometriosis	Zhejiang University, Hangzhou, China	2022	Granted
CN116531344	A macrophage membrane-modified nanoparticle and its preparation method and application	PLGA, PFH and SPIO	Myocarditis	Children’s Hospital of Chongqing Medical University, Chongqing, China	2023	Filed
CN117776276	Iron oxide nanoparticle and application thereof in magnetic particle imaging	Iron oxide	Imaging tracer agent	Xidian University, Xi’an, China	2023	Filed
CN117838655	Bionic nanoparticle for targeted therapy of monkey pox virus and blocking propagation of monkey pox virus as well as preparation method and application of bionic nanoparticle	ICG and DPT	Monkey pox virus	Dermatology Hospital of Southern Medical University, Guangzhou, China	2023	Filed
CN116637082	Heart resident macrophage-derived bionic nano system and preparation and application thereof	Exosomes	Heart	Dongguan People’s Hospital, Dongguan, China	2023	Filed
CN117562987	Universal mucosal vaccine vector and preparation method thereof	Poly	Animal mycoplasma infection	Jiangsu Academy of Agricultural Sciences, Nanjing, China	2023	Filed
CN117244059	A hybrid membrane-coated CuS composite nanoparticle and its preparation method and applications	CuS and MnO	Cancer	Xuzhou Medical University, Xuzhou, China	2023	Filed
CN117731636	PLGA and macrophage membrane-coated emodin nanoparticles capable of fluorescence detection, their preparation method and applications	PLGA	-	Chongqing Medical and Pharmaceutical College, Chongqing, China	2023	Filed
CN116407615	Macrophage membrane-coated antimicrobial peptide nanoparticles for the development and preparation of broad-spectrum antibacterial drugs for the treatment of bacterial sepsis	-	Septic/bacterial infection	Wenzhou Institute UCAS, Wenzhou, China	2023	Filed
CN116270533	Application of PCOD585-loaded nanoparticles coated with macrophage membranes in myocardial ischemia-reperfusion injury	PLGA	Myocardial ischemia-reperfusion injury	Zhongshan Hospital Affiliated to Fudan University, Shanghai, China	2023	Granted
CN119679756	Macrophage membrane-coated nanoparticles loaded with TRAF7 and their application in non-alcoholic fatty liver disease	PLGA	Nonalcoholic fatty liver dease	Chongqing Medical University, Chongqing, China	2024	Filed
CN118320115	Macrophage membrane coated polymyxin E acid-sensitive nano-drug as well as preparation method and application thereof	Polymyxin E acid-sensitive nano-drug	Gram-negative bacterium	Xinxiang Medical University, Xinxiang, China	2024	Filed
CN118252942	Cell membrane bionic preparation with sponge factor effect as well as preparation method and application of cell membrane bionic preparation	Black phosphorus	Alzheimer’s disease	Guangzhou University of Chinese Medicine, Guangzhou, China	2024	Filed
CN118236508	A macrophage membrane-coated copper-based metal-organic framework and its preparation method	CuBN	Antibacterial properties	Guangxi Medical University, Guangxi, China	2024	Filed
CN118787608	A liver inflammation-targeted nanoparticle loaded with the TRPV1 agonist capsaicin, and its preparation method and applications	PLGA	Cholestasis	University of Electronic Science and Technology of China, Chengdu, China	2024	Filed
CN118976006	An activated macrophage membrane-encapsulated miRNA nanodelivery system and its preparation method	ZIF-8	Targeted delivery	The First Affiliated Hospital of Soochow University, Suzhou, China	2024	Filed
CN119015256	A vitamin C-loaded macrophage membrane biomimetic nanogel and its preparation method and applications	Nano hydrogel	Tissue defect repair	Nanjing Drum Tower Hospital, Nanjing, China	2024	Filed
CN119950450	A macrophage membrane-coated nanocomposite, its preparation method and applications	LDH	Myocardial ischemia-reperfusion injury	Tongji University, Shanghai, China	2025	Filed

*AuNPs Gold nanoparticles; *PCM* PBAP-CDI-Mannose; *PLGA* Polylactic acid-glycolic acid copolymer; *PFH* Perfluorohexane; *SPIO* Superparamagnetic iron oxide; *LDH* Layered double hydroxides; *ICG* Indocyanine green; *DPT* Layered double hydroxides

Collectively, these patents document the evolution of MΦ-NPs from proof-of-concept studies to translational frameworks, outlining a clear trajectory toward clinical application. The growing number and diversity of patents underscore both the rapid technological maturation and the immense clinical potential of MΦ-NPs in addressing inflammatory diseases. Improvements in membrane stability, enhanced targeting precision, and scalable production processes are converging to form the technological foundation for future clinical implementation.

## Toxicity research and safety assessment of NPs

Toxicity assessment represents a fundamental regulatory requirement in the biomedical application of nanomaterials. While nanotechnology has catalyzed major breakthroughs in targeted drug delivery and diagnostic innovation, potential toxicity risks remain a major concern. Safety evaluations are generally divided into two major categories: in vitro and in vivo. In vitro assays typically include cytotoxicity and apoptosis testing, while in vivo assessments involve biodistribution analysis, clearance rate measurement, hematological profiling, histopathological examination, and evaluation of organ-specific toxicity such as hepatic, renal, pulmonary, and dermal effects [97, 98]. Vinay Kumar et al. [99] provided a comprehensive summary of recent research progress regarding the chemical degradation mechanisms and toxicological behavior of nanomaterials (Table 4).

**Table 4:** Toxicity assessment techniques for various functional materials

Sr no.	D rug carriers	Toxicity assessment techniques/models	Toxic effect
1	Carbon-based nanocarriers	Bacteria, microalgae, crustacean, zebrafish, *D. melanogaster*	Metabolic activity
2	Gold-based nanocarriers	Guinea pig, mouse, rat	Cell viability
3	Silver-based nanocarriers	Atrocytes	Mitochondrial damage
4	Quantum dots	Breast cancer cells	ROS production
5	Iron-based nanoparticles	Human hepatocytes	Actin filament integrity
		Human bronchial epithelial cells	Blood − brain barrier destruction
		Messenchymal stem cells	Alteration of gene
6	Silicon-based nanoparticles	Lung tissues	-
		Human skin	-

Table adapted from Vinay Kumar et al. [99]

Importantly, MΦ-NPs exhibit a distinct safety advantage over conventional nanomaterials by effectively mitigating inherent toxicity risks. The macrophage membrane functions as a natural biological interface that minimizes immune recognition and clearance, thereby enhancing systemic tolerance. Furthermore, the lipid and protein components of the membrane reduce non-specific cellular interactions and suppress cytotoxicity, immunogenicity, and off-target accumulation.

This biomimetic protective mechanism confers a fundamental reduction in toxicity risk compared with unmodified or chemically modified nanoparticles. Consequently, macrophage membrane coating not only enhances biocompatibility but also strengthens the translational safety profile of nanomedicines. Establishing standardized toxicity evaluation protocols and long-term biocompatibility studies will be crucial to further advance MΦ-NPs toward clinical adoption.

## Summary and prospect

This review has systematically summarized the recent advances in MΦ-NPs as therapeutic platforms for inflammatory diseases. Compelling evidence from diverse preclinical models, including infectious, autoimmune, and metabolic inflammatory disorders, has demonstrated the efficacy, adaptability, and translational potential of MΦ-NPs in modulating complex inflammatory responses.

Despite these encouraging findings, the clinical translation of MΦ-NPs remains limited due to a series of unresolved scientific and technical barriers. Major challenges include the low efficiency and reproducibility of membrane extraction, inter-batch variability in coating consistency, potential immunogenicity arising from retained surface proteins, and the long-term biosafety risks associated with chronic in vivo accumulation. Addressing these bottlenecks requires not only technological refinement but also the establishment of standardized quality control and manufacturing protocols that align with regulatory expectations for biomedical products.

At present, the development of robust and standardized membrane isolation and modification procedures represents a key prerequisite for clinical translation. Integrating computational tools such as molecular dynamics simulations to predict membrane–cell interactions and assess toxicity risks may further enhance process predictability and design precision. Importantly, GMP-scale membrane separation technologies—widely applied in biologics manufacturing—offer a feasible pathway toward large-scale MΦ-NPs production. Through controlled processes such as microfiltration, ultrafiltration, nanofiltration, and reverse osmosis, these systems can ensure batch-to-batch reproducibility, aseptic operation, and contaminant removal. Together, these measures provide a compliant and scalable foundation for advancing MΦ-NPs from laboratory research to clinical-grade production.

In addition to process-related challenges, broader nanomedicine-specific limitations must also be addressed. Issues such as inherent material toxicity, rapid hepatic and renal clearance, and suboptimal systemic bioavailability continue to restrict clinical applicability. Overcoming these obstacles will require systematic optimization of nanoparticle synthesis, surface functionalization, and formulation design to enhance safety, pharmacokinetics, and therapeutic precision.

Furthermore, the clinical scope of MΦ-NPs remains to be expanded beyond current preclinical models. Their application in complex autoimmune diseases such as systemic lupus erythematosus and multiple sclerosis remains largely unexplored. Extending investigations to these underrepresented pathologies will help establish a more comprehensive understanding of MΦ-NPs across diverse inflammatory contexts and disease stages.

In conclusion, MΦ-NPs—constructed through the rational integration of functional nanocores, engineered macrophage membranes, and optimized core–shell assembly—demonstrate remarkable capabilities in targeted delivery, immune modulation, and systemic biocompatibility. As advances in nanotechnology, immunology, and computational biodesign converge, these biomimetic systems are poised to redefine therapeutic paradigms for inflammatory diseases. With continued innovation and regulatory harmonization, MΦ-NPs hold the potential to drive the next generation of precision anti-inflammatory therapies with enhanced efficacy, safety, and translational feasibility.

## Data Availability

No datasets were generated or analysed during the current study.

## Abbreviations

MΦ-NPs: Macrophage membrane-coated nanoparticles
TNF-α: Tumor necrosis factor-alpha
IL-6: Interleukin-6
VEGF: Vascular endothelial growth factor
NSAIDs: Non-steroidal anti-inflammatory drugs
COX-2: Cyclooxygenase-2
NPs: Nanoparticles
PLGA: Polylactic acid-glycolic acid copolymer
DLS: Dynamic light scattering
TEM: Transmission electron microscopy
COVID-19: Coronavirus disease 2019
UPNCs: Upconversion nanoparticles
FLI: Fluorescence imaging
*C. albicans*: *Candida albicans*
RA: Rheumatoid arthritis
IBD: Inflammatory bowel disease
AS: Atherosclerosis
RBCs: Red blood cells
